# Real-World Experience of L-Glutamine in Sickle Cell Disease: A Retrospective Observational Study

**DOI:** 10.3390/pharmacy13030084

**Published:** 2025-06-13

**Authors:** Shouq Turkistani, Atika AlHarbi, Mansoor Khan, Aeshah AlAzmi, Sultan Almutairi, Naglla Elimam, Sultan Alotaibi

**Affiliations:** 1 Department of Pharmaceutical Care Clinical Services, Ministry of National Guard Health Affairs (MNGHA), Jeddah 21423, Saudi Arabia; Shouq.turkistani@gmail.com (S.T.); alharbiat@mngha.med.sa (A.A.); khanma1@mngha.med.sa (M.K.); alazmiai@mngha.med.sa (A.A.); 2 King Abdullah International Medical Research Center, Jeddah 21423, Saudi Arabia; almutairisu1@mngha.med.sa (S.A.); elimamna@mngha.med.sa (N.E.); 3 King Saud bin Abdulaziz University for Health Sciences, Jeddah 21423, Saudi Arabia; 4 Adult Medical Hematology/Oncology/BMT Department, Ministry of National Guard Health Affairs (MNGHA), Jeddah 21423, Saudi Arabia; 5 Pediatric Hematology/Oncology/BMT Department, Ministry of National Guard Health Affairs (MNGHA), Jeddah 21423, Saudi Arabia; 6 Department of Pharmaceutical Care Clinical Services, Ministry of National Guard Health Affairs (MNGHA), Taif 26573, Saudi Arabia; Otibisu@mngha.med.sa

**Keywords:** sickle cell disease (SCD), L-glutamine, Endari, vaso-occlusive crises (VOCs), clinical meaningfulness, cost effectiveness, hydroxyurea, hematology, real-world experience

## Abstract

Sickle cell disease (SCD) affects millions globally, with approximately 0.26% of the Saudi population impacted. Despite standard treatments, patients frequently experience vaso-occlusive crises (VOCs). This retrospective observational study evaluated the real-world effectiveness of L-glutamine (Endari^®^) in reducing SCD-related complications in the Saudi population, where data remain limited. Patients aged five and older who received L-glutamine from June 2019 to June 2023 were included. The primary endpoint was VOC frequency through week 48. Descriptive statistics and paired t-tests compared outcomes before and after treatment. Fifteen patients (median age 12 years, 53% female) met the inclusion criteria; all were on maximum tolerated hydroxyurea. Eleven completed 48 weeks, showing a median VOC reduction from 4 to 3 (*p* = 0.44). Hospital stay duration remained unchanged (median 7 days, *p* = 0.72). Laboratory parameters were largely stable, except for a 61.9% increase in reticulocyte count (*p* = 0.03). The estimated annual treatment cost exceeded SAR 2 million (USD ~547,840). L-glutamine did not produce statistically significant improvements in VOC frequency, though numerical trends were observed. Given the small sample size and limited statistical power, the findings are exploratory. Larger, well-powered, multicenter studies are needed to confirm L-glutamine’s potential benefits in this population.

## 1. Introduction

Sickle cell disease (SCD) is a genetic blood disorder caused by the inheritance of two abnormal beta-globin genes, at least one of which carries the sickle mutation (HbS). Different genotypes include HbSS or HbS/β-thalassemia [1,2]. SCD affects millions globally; based on the ministry of health in Saudi Arabia, 4.2% of the population has the trait, and 0.26% has SCD, with the most significant prevalence in the Eastern province [3,4].

In patients with SCD, this hemoglobin (Hb) gene defect leads to serious and life-threatening consequences such as inflammation, hemolytic anemia, splenic dysfunction resulting in impaired immunity to encapsulated organisms, vascular occlusion, and pain, all of which have an adverse impact on the patient’s health-related quality of life (HRQOL). Stroke, skin ulcers, priapism, acute and chronic organ damage, and a shortened life expectancy are possible secondary complications [5,6].

Pain is the most prevalent consequence of SCD and the most common reason for emergency room visits among SCD patients [5]. They continue to suffer from multiple painful vaso-occlusive crises (VOCs), resulting in hospitalizations and end-organ damage, despite using blood transfusions, hydroxyurea, and opioids as cornerstones in managing SCD-related complications in children and adults [5,6]. The complex and poorly understood pathophysiology of SCD poses challenges to managing patients, both acutely and chronically [6]. New targeted medications have been developed to address SCD in response to emerging pathophysiological insights [7]. Over thirty pharmaceutical agents are currently under investigation for treating and preventing SCD-related complications, three of which have recently received FDA approval targeting vaso-occlusion and HbS polymerization: L-glutamine, crizanlizumab, and voxelotor [7,8]. However, recent regulatory updates have affected their availability: the conditional marketing authorization for crizanlizumab was revoked in August 2023 [9], and voxelotor was withdrawn from the market by Pfizer in 2024 due to safety concerns [10].

In SCD patients, oxidative stress plays a central role in the pathophysiology of hemolysis, inflammation, and vaso-occlusion. The Nicotinamide Adenine Dinucleotide (NAD) redox potential serves as a key indicator of oxidative stress by reflecting the balance between oxidized and reduced NAD molecules. Glutamine, an L-α-amino acid, is one of the most abundant amino acids in the body. Due to the accelerated red blood cell (RBC) turnover caused by chronic hemolysis in SCD, the demand for glutamine increases, making it a conditionally essential amino acid in this context. The proposed therapeutic rationale for L-glutamine supplementation in SCD lies in its antioxidant properties. L-glutamine supports the synthesis of antioxidants such as reduced glutathione, NAD(H), NADP(H), and nitric oxide, thereby enhancing redox balance and reducing oxidative damage [11,12]. Clinical and preclinical evidence indicates that L-glutamine increases NAD redox potential and NADH levels in sickle RBCs, potentially decreasing oxidative susceptibility and contributing to clinical improvements [13,14,15,16]. In a phase 3 clinical trial, L-glutamine was shown to reduce the frequency of vaso-occlusive crises by 25%, hospitalizations by 30%, and acute chest syndrome episodes over 48 weeks in both pediatric and adult SCD patients, with or without hydroxyurea [17]. Consequently, L-glutamine (Endari^®^) became the first therapy approved for SCD treatment since hydroxyurea in 1998 [18].

Current data regarding the use and effectiveness of L-glutamine for sickle cell disease (SCD) in the Saudi population remain scarce. This knowledge gap limits evidence-based treatment strategies and optimal outcomes for these patients. Despite national screening programs, substantial gaps remain in access to disease-modifying therapies, comprehensive care centers, and patient education. Recent local studies also highlight the limited availability of newer agents such as L-glutamine, and regional disparities in hydroxyurea use remain a concern [19]. Findings from the Real-World Assessment Survey for SCD in Saudi (ROARS) revealed significant gaps in the management of sickle cell disease by non-specialist healthcare providers, with limited use of newer therapies such as L-glutamine and marked variability in treatment approaches across regions [20].

Moreover, our hospital requested L-Glutamine for 22 patients on a non-formulary basis until November 2022. There was a huge cost impact on our organization’s stretched budget. In addition to that, L-Glutamine has not yet been approved by the Saudi Food and Drug Authority (SFDA), and the procurement of unregistered medication is a significant challenge in Saudi Arabia. Staying up to date with drug regulations and compliance requirements is essential, as reported by a couple of groups of formulary management experts in Saudi Arabia [21,22]. This prompted our Pharmacy and Therapeutic (P&T) Committee to initiate a real-world evidence study on the effectiveness of L-Glutamine therapy in SCD patients to support their formulary decision of addition vs. rejection of the drug for its use in these patient population. To address this need, our study aimed to evaluate both the effectiveness and appropriateness of L-glutamine therapy in reducing SCD-related complications among adult and pediatric patients treated at King Abdulaziz Medical City, Jeddah.

## 2. Materials and Methods

Study design and patients:

We conducted a retrospective, observational, single-center study at King Abdulaziz Medical City in Jeddah, Saudi Arabia, between June 2019 and June 2023. This facility is one of the largest tertiary care centers, where a significant number of pediatric and adult patients with SCD are referred to the hematology section for disease management. Our study included those who had SCD and were actively receiving hydroxyurea prior to initiating L-Glutamine (Endari^®^).

L-Glutamine was initiated for patients who were at least 5 years old and had documented at least two crises in the previous year, with no upper limit on the number of crises, despite the use of hydroxyurea. We used the standard dosing as per the FDA label [<30 kg: 5 g (1 packet) twice daily (total dose 10 g/day), 30 to 65 kg: 10 g (2 packets) twice daily (total dose 20 g/day), >65 kg: 15 g (3 packets) twice daily (total dose 30 g/day)] [23]. All patients were regularly followed up in hematology clinics with a follow-up period of 24 and 48 weeks. Study participants who were lost to follow-up after treatment initiation, as determined from their medical records, were excluded from the analysis.

Endpoints:

The primary endpoint was to determine the frequency of VOCs in sickle cell disease patients through week 48 after L-glutamine initiation and compare it to historical control for 48 weeks prior to L-glutamine initiation.

Secondary endpoints included identifying the frequency and length of hospital stay due to VOCs and evaluating the percentage of laboratory parameter changes (hemoglobin level, hematocrit level, reticulocyte count, and lactate dehydrogenase (LDH) level) from baseline through weeks 24 and 48 and the cost impact of the use of L-glutamine.

Data collection:

For the purpose of this study, a data collection tool was developed after a thorough review of the symptoms acquired from each patient’s medical record at baseline and at weeks 24 and 48. Data on patient demographics (age, gender, weight), diagnosis using WHO ICD version 10, hydroxyurea use, laboratory parameters (hemoglobin levels, hematocrit, reticulocyte count, LDH levels), type of VOC and its frequency, length of hospital stay, and treatment discontinuation were collected. Clinical parameters, such as VOC type, VOC frequency, and duration of hospital stay, were collected 48 weeks before therapy commencement (referred to as historical control).

Definition:

VOCs were defined according to Niihara et al. as acute pain episodes for no apparent cause other than a vaso-occlusive event involving a medical facility visit and being treated with oral or parenteral narcotic agents or a nonsteroidal anti-inflammatory drug. In addition, the occurrence of chest syndrome (chest-wall pain in association with findings of a new pulmonary infiltrate on chest X-ray films and fever), priapism, and splenic sequestration are considered sickle cell crises even if the symptoms are not painful enough to require narcotics [17]. Secondary endpoints included determining the frequency of VOCs in sickle cell disease patients through week 24 after L-glutamine initiation, identifying the length of hospital stay due to VOCs after starting L-glutamine through week 48, and evaluating the percentage of laboratory parameter changes (hemoglobin level, hematocrit level, reticulocyte count, and LDH level) from baseline through weeks 24 and 48. For the cost impact, prices of L-glutamine were obtained from the National Unified Procurement Company (NUPCO) in the public sector.

Statistical analysis:

Descriptive statistics were calculated to summarize the demographic and clinical characteristics of the study population, including percentages, the mean ± standard deviation (SD) for normally distributed data, and the median [interquartile range, IQR] for non-normally distributed data. To compare the effects of L-glutamine and historical control, a paired t-test was utilized. Two-tailed statistical significance was indicated by a *p* value of <0.05. The statistical program KNIME Analytics was used for all statistical analyses.

A post hoc power analysis was conducted for the primary outcome (VOC frequency at 48 weeks). Assuming a mean difference of 1 VOC event, an SD of 1.5, α = 0.05, and *n* = 11, the estimated power was approximately 38%.

## 3. Results

Patients:

A total of 22 SCD patients were identified initially, and L-glutamine was requested for all 22 patients. However, after following the predefined inclusion criteria, only 15 patients were found to be eligible and were included in the final analysis (7 adults and 8 pediatric patients). The excluded patients comprised five individuals who had not initiated L-glutamine treatment at the time of data collection and two patients who had received the medication for less than 24 weeks.

Among the 15 patients included in the analysis, 11 completed the entire 48-week study duration. The reasons for incomplete follow-up varied, including loss of follow-up, unavailability of the medication, non-compliance, or insufficient information documented in their medical records. More than half of the patients were females (8, 53%), with a median age of 12 (IQR 7–20) years old and an average weight of 34.5 ± 16.6. All patients were on hydroxyurea before L-glutamine initiation and had reached their maximum tolerated dose, with a median of 24 (IQR 20–30) mg/kg (Table 1). All of them continued using both hydroxyurea and L-glutamine together, except for one adult patient who was only on L-glutamine due to intolerance to hydroxyurea.

**Figure 1:**
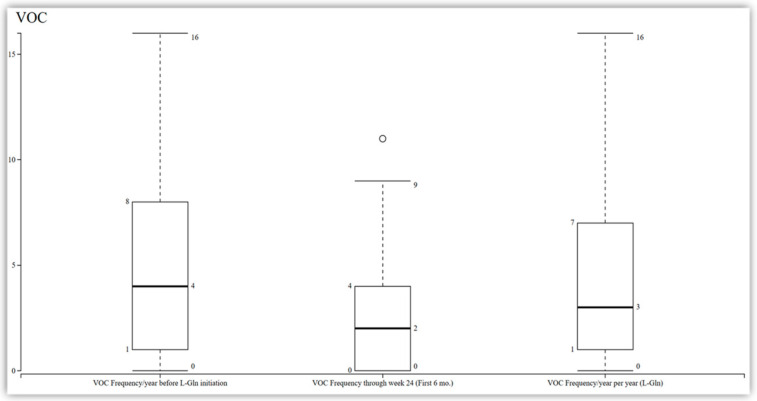
VOC frequency before and after L-Gln initiation in patients with sickle cell disease (N = 15; n = 11 at 48 weeks). Median VOCs decreased from 4 events/year at baseline to 2 events at 24 weeks and 3 events at 48 weeks. This reduction was not statistically significant. Boxes represent the interquartile range (IQR), horizontal lines within boxes indicate medians, and whiskers show the full range. VOC: vaso-occlusive crisis. L-Gln: L-glutamine.

**Figure 2:**
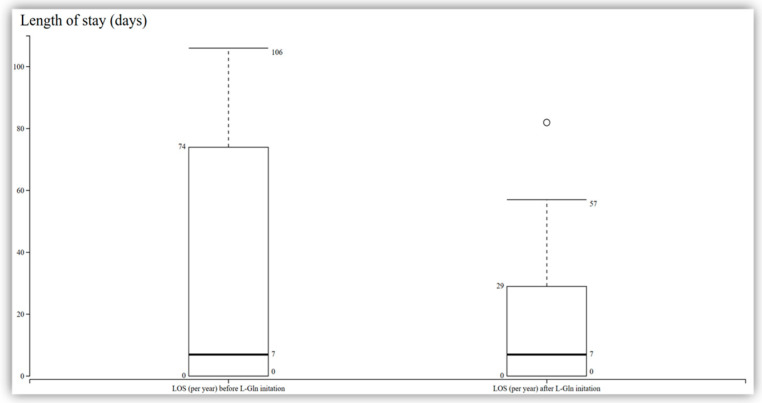
LOS before and after L-Gln initiation in patients with sickle cell disease (N = 15; n = 11 at 48 weeks). Median LOS remained 7 days/year both before and after treatment, with the interquartile range narrowing from 0–74 days to 0–29 days. This change was not statistically significant. Boxes represent the interquartile range (IQR), horizontal lines within boxes indicate medians, and whiskers show the full range. LOS: length of stay. L-Gln: L-glutamine.

Clinical results:

Among the 11 patients who completed the 48-week study, a reduction in the frequency of vaso-occlusive crises was observed compared to their baseline. However, this reduction did not reach statistical significance, with a median change from 4 to 3 (95% CI −1.37 to 0.65; *p* = 0.44) at 48 weeks and a median change from 4 to 2 at 24 weeks (Figure 1). Additionally, the analysis of the data revealed that there was no significant change in the length of stay among the patients. The median length of stay was observed to be seven days (IQR 0–29; *p* = 0.72) (Figure 2).

Laboratory results:

The laboratory parameters were assessed at weeks 24 and 48. The results indicate that there were no significant differences in these parameters compared to the baseline levels at both time points. For hemoglobin, the baseline median was 8.7 g/dL (IQR 8.4–9.8), with a 2.3% change at week 24 (IQR 8.4–9.8; *p* = 0.84) and a 4.6% change at week 48 (IQR 8.8–10; *p* = 0.49). However, noteworthy increases were observed in one specific laboratory parameter. At week 48, a substantial 61.9% rise in the reticulocyte count was observed, with a median of 278.5 (IQR 153–299; *p* = 0.03) compared to baseline (Table 2).

Cost Impact:

The prices of L-glutamine were obtained from NUPCO in the public sector. The unit price of L-glutamine 5 g/sachet is SAR 85.28 (22.74 USD). We used all 22 patients for the cost impact since L-glutamine was requested for all 22 patients on a non-formulary basis. The median dose used in pediatrics (n = 11) was 5 g per oral (PO) twice daily, and the median dose used in adults (n = 11) was 10 g PO twice daily. The cost of therapy for one pediatric patient per year was SAR 62,255 (16,601 USD), and the cost of therapy for one adult patient per year was SAR 124,509 (33,202 USD). The total estimated cost of therapy for 22 patients per year was SAR 2,054,399 (547,840 USD). This is not a cost-effectiveness modeling study; rather, it is just providing a cost impact of utilization of L-glutamine. It also anticipates cost savings or cost avoidance by rejecting the drug for its utilization in SCD patients in our center. Our P&T committee rejected the drug for this indication, and hence, cost savings or cost avoidance is important to mention here.

## 4. Discussion

Sickle cell disease is a prevalent condition with significant health implications for both adult and pediatric patients. Despite the high prevalence of SCD, there is a notable lack of studies investigating the efficacy and appropriateness of L-glutamine in reducing SCD-related complications. Hence, the aim of this study was to fill this gap by evaluating the efficacy and appropriateness of L-glutamine.

The results from our study, along with previous research on L-glutamine in SCD patients, consistently demonstrate a reduction in the frequency of VOCs. Although our study showed only a trend toward a reduction in VOC frequency that did not reach statistical significance, this finding aligns with the results from Niihara et al. (2018) and Elenga et al. (2022) [17,24], which observed similar trends. Notably, unlike those studies, our results did not achieve statistical significance. Niihara et al. conducted a phase 3 trial and showed a significant reduction in pain crises by 25% in SCD patients treated with L-glutamine. Similarly, Elenga et al.’s study on pediatric and adult SCD patients revealed significant improvements in clinical outcomes, including decreased pain crises, hospitalizations, days of hospitalization, blood transfusions, and acute chest syndrome events following L-glutamine therapy.

Importantly, the lack of statistical significance in our results may be attributed to the study’s limited statistical power. A post hoc power analysis revealed that the study had only 38% power to detect a one-event reduction in VOC frequency at 48 weeks, which is well below the conventional 80% threshold. This further underscores the exploratory nature of our findings and the need for caution in interpreting the absence of statistically significant results.

One other vital aspect to consider is the impact of L-glutamine on the length of hospital stays. We found that L-glutamine did not significantly affect the duration of hospital stays among patients receiving this treatment. These findings contrast with some previously published studies that have reported a reduction in hospitalizations and days of hospitalization with L-glutamine therapy in SCD patients. For example, Elenga et al. demonstrated a decrease in hospitalizations and days of hospitalization in SCD patients receiving L-glutamine [24]. as Also, Niihara et al. reported a reduction in hospitalizations compared to the placebo group [17].

In terms of laboratory results, most parameters did not show significant differences compared to baseline levels at both weeks 24 and 48. However, one laboratory parameter exhibited noteworthy change. Greater reticulocytosis in SCD patients emphasizes the disease’s hallmark of chronic peripheral hemolysis [25]. The pathogenesis of SCD may be influenced by the distinct morphological and adhesive properties of sickle reticulocytes [26]. Our study showed a significant 61.9% rise in the reticulocyte count at week 48 compared to baseline, which contradicts the results of other studies. This unexpected increase in the reticulocyte count may point to more hemolysis and not less. We found that two patients, one adult and one pediatric SCD patient, underwent splenectomy. These two patients had significant elevations in reticulocyte counts reaching 582 in the pediatric patient and 390 in the adult patient. Reticulocytosis is a common phenomenon observed after splenectomy which is not accompanied by significant changes in cell volume. Hence, reticulocytosis after splenectomy does not necessarily indicate a stress-induced erythropoietic stimulus [27]. Moreover, we had three more patients with frequent VOCs whose reticulocyte count was significantly elevated, with the maximum retic count reaching 504, 450, and 426 in these three patients. These patients had not shown any response to L-glutamine. Moreover, 5 out of 11 patients with significantly elevated retic counts resulted in a 61% increase in retic count in the total study population, which is influenced by the small sample size.

The drug monograph of L-glutamine, along with the result of this study, was presented to the P&T committee on 28 February 2023. The P&T committee rejected the addition of L-glutamine to the formulary because of the lack of effectiveness and even recommended against non-formulary use of this drug in SCD patients. Hence, this study has a cost avoidance impact of around SAR 2,054,399 (547,840 USD) per year for 22 patients, and total cost savings over the 18 months have been SAR 3,081,599 (821,760 USD) since the P&T committee circulated minutes of the meeting in March 2023 stating the rejection of the drug. This cost avoidance is expected to be even more as our hospital did not request the drug for any new patient after the P&T made their decision. This paper was presented on the Saudi Commission for Health Specialists research day in 31 August 2023, and the data were shared publicly. This study has a significant impact on avoiding unnecessary costs associated with the use of this drug when it comes to formulary consideration of the drug nationally.

When comparing our findings to previously published data, it is crucial to acknowledge the limitations of our study. First, the retrospective design and the limited availability of complete data may affect our conclusions. Second, the relatively small sample size reduced the statistical power to detect significant differences, as confirmed by our post hoc analysis. Additionally, we were unable to directly assess the impact of disease severity and specific genetic polymorphisms on the efficacy of L-glutamine due to data constraints. Furthermore, we could not retrieve compliance data, as we did not have direct interactions with the patients. Finally, the reliance on a historical control group may introduce temporal confounding, potentially impacting the study’s validity. This heterogeneity among SCD patients, which includes variations in disease severity, concurrent treatments, and genetic differences, may have contributed to the variability in outcomes observed.

Further studies with larger, multicenter cohorts and detailed evaluations of clinical and genetic factors are warranted to validate our findings. Future research should also include patient-reported outcomes, such as pain scores and quality of life measures, as well as long-term safety assessments, to provide a more comprehensive evaluation of L-glutamine’s efficacy and safety in SCD patients.

## 5. Conclusions

L-glutamine treatment in our cohort of sickle cell disease patients did not result in statistically significant improvements in the frequency of VOCs, although some numerical trends were observed. Given the small sample size and limited statistical power, these findings should be interpreted as exploratory rather than definitive. Further well-powered, multicenter studies are warranted to validate the potential benefits of L-glutamine in this patient population.

## Figures and Tables

**Table 1:** Baseline characteristics.

Characteristics	Patients with SCD Who Received L-Glutamine N = 15
Age (Y), median [IQR]	12 [7–20]
Gender, n (%) - Male - Female	- 7 (47%) - 8 (53%)
Weight (kg), mean ± SD	34.5 ± 16.6
MTD of Hydroxyurea (mg/kg), median [IQR]	24 [20–30]

SCD: sickle cell disease. MTD: maximum tolerated dose.

**Table 2:** Laboratory parameters.

Lab	Baseline N = 15	Week 24 N = 15	Percentage Change %	(95% CI) *p* Value	Week 48 N = 11	Percentage Change %	(95% CI) *p* Value
LDH U/L	400.5 [329–480]	494 [341–655]	23.3	(−188.45–−2) 0.05	418 [315.5–438.5]	4.4	(−74.14–63.14) 0.84
Hemoglobin g/dL	8.7 [8.4–9.8]	8.9 [8.4–9.8]	2.3	(−0.62–0.51) 0.84	9.1 [8.8–10]	4.6	(−1.08–0.55) 0.49
Reticulocyte × 10^9^/L	172 [133–206]	188 [153–238]	9.3	(−98.56–27.92) 0.24	278.5 [153–299]	61.9	(−160.53–−11.58) 0.03
Hematocrit %	27.6 [25.3–29.4]	28 [25.8–31.5]	1.4	(−2.42–1.07) 0.42	27.7 [27.1–30.4]	0.4	(−3.20–1.49) 0.44

Data presented as median [IQR]. LDH: lactate dehydrogenase. CI: confidence interval.

## Data Availability

The data are contained within the article.

## Abbreviations

The following abbreviations are used in this manuscript:

CI	Confidence Interval
FDA	Food and Drug Administration
Hb	Hemoglobin
HbS	Hemoglobin S
HRQOL	Health-Related Quality of Life
IQR	Interquartile Range
LDH	Lactate Dehydrogenase
LOS	Length of Stay
NAD	Nicotinamide Adenine Dinucleotide
NUPCO	National Unified Procurement Company
PO	Oral
RBC	Red Blood Cell
SCD	Sickle Cell Disease
SFDA	Saudi Food and Drug Administration
SD	Standard Deviation
VOC	Vaso-Occlusive Crisis
P&T	Pharmacy and Therapeutic
